# Study on Seed Emergence and Seedling Growth of *Artemisia Desertorum* with Superabsorbent Polymers

**DOI:** 10.3390/polym12122873

**Published:** 2020-11-30

**Authors:** Wenxu Zhang, Qian Liu, Shengfang Liu, Jing Chen, Lulu Guo, Peng Wang, Ziqiang Lei

**Affiliations:** 1 Key Laboratory of Eco-functional Polymer Materials of the Ministry of Education, Northwest Normal University, Lanzhou 730070, China; Liuq36@163.com (Q.L.); l18390243983@163.com (S.L.); chenjing_2906@163.com (J.C.); gll961218@163.com (L.G.); wangpeng19960202@163.com (P.W.); leizq@nwnu.edu.cn (Z.L.); 2 College of Chemistry and Chemical Engineering, Northwest Normal University, Lanzhou 730070, China; 3 Key Laboratory of Eco-environmental Polymer Materials of Gansu Province, Northwest Normal University, Lanzhou 730070, China

**Keywords:** superabsorbent polymer, drought resistance, *Artemisia desertorum*, seed emergence, seedling growth

## Abstract

In this study, the emergence rate, emergence potential, root length, stem length, water consumption, and biomass around a root system were used as evaluation indexes, and we used the laboratory-prepared super absorbent resin watermelon rind (WMR)-p (AA–*co*–DAAM) (superabsorbent polymer 1—SAP_1_), WMR-p (AA–*co*–DAAM)/palygorskite (PGS) (SAP_2_) in a laboratory and commercially available water-retaining agents (SAP_RX_ and SAP_HDB_) to assist the emergence of *Artemisia* *desertorum* seeds and seedling growth; then, their water absorption and thermal stability were discussed. The results showed that the thermal stability of an SAP prepared in the laboratory and the water consumption during seed emergence, root length, and stem length after emergence were better than those of an SAP purchased on the market, and this information could help to promote the emergence and seedling growth of *Artemisia* *desertorum*. While enhancing the drought resistance of plants, using a laboratory-produced SAP can effectively reduce the number of artificial irrigations.

## 1. Introduction

The total area of arid and semi-arid areas in China has reached 4.55 million hm^2^, accounting for 47% of the total land area of China [1]. Especially in northwest China, where drought and water shortages are serious, the desertification area accounts for 80% of the national area [2]. Moreover, a long period of spring drought resulted in the failure of plant seeds to germinate and grow due to water shortage. Therefore, resisting drought and save water and make full use of rainfall is the key to solve this problem, as well as the key to the sustainable development of desertification prevention and control in northwest China.

*Artemisia desertorum* is a kind of drought-tolerant, perennial herbaceous plant that grows in desert and semi-desert areas of China [3,4]. It has strong and developed roots, and most of its stems are tufted. It has a good role in preventing wind and sand [5]. Its seeds sometimes cannot germinate and grow in time due to drought in desert areas, and the presence of a high absorbent resin could provide water to maintain emergence and seedling growth.

Superabsorbent polymers (SAPs) are kinds of three-dimensional crosslinked hydrophilic polymers that can absorb and retain large amounts of water even under a high pressure [6]. A superabsorbent polymer contains a large number of hydrophilic groups that can absorb at water tens to thousands of times of its weight and swell into a gel-like form [7]. It is non-toxic and harmless, and it has applications in various fields such as personal hygiene products [8], medical materials [9], drug release [10], building materials [11], wastewater treatment [12], fire extinguishing agent [13], and agriculture, forestry, and horticulture [14,15]. In agriculture, the application of SAPs can significantly reduce the frequency of irrigation, greatly improve soil water retention capacity, enhance plant survival rates, reduce pesticide residues, and improve microbial activity in soil [16]. SAPs can also be used as seed coatings and seed dressings [17], and applying them to soil can not only improve the soil by increasing soil fertility [18,19], maintaining soil moisture, and acting as "mini reservoirs" for plants [20], they can also assist the emergence and growth of plants in arid desert areas [21,22]. However, different preparation methods, preparation conditions, preparation raw materials, and production formulas can cause differences in the performances of superabsorbent resin in various aspects [23].

Therefore, the main content of this study is to take watermelon rind (WMR) as raw material, WMR-p (AA–*co*–DAAM) (SAP_1_) and WMR-p (AA–*co*–DAAM)/palygorskite clay (PGS) (SAP_2_) super absorbent resins were prepared as the research objects. The performances of water absorption, water retention, and thermal stability were evaluated. The effects of laboratory-made superabsorbent resins on the seed emergence and seedling growth of *Artemisia desertorum* were pinpointed.

## 2. Materials and Methods

### 2.1. Preparation of Materials

SAP_1_ and SAP_2_ were made in the laboratory, and two different water-preserving agents, Runxing (Yantai, China) and Handibao (Weifang, China), were purchased from the market.

Prior to synthesizing the superabsorbent polymer, WMR and PGS were pretreated. After being freeze-dried, the watermelon rind was crushed and sifted to obtain PGS powder.

PGS was processed in two steps. In the first step, distilled water was added to PGS powder and mixed evenly through mechanical agitation. After centrifugation at a speed of 8000 r/min for 5 min, the precipitate was washed multiple times with distilled water and fully dried in an oven at 70 °C. In the second step, the dried PGS was prepared into a powder, poured into a three-necked flask with a sufficient ratio of 1 mol/L HCl (mass ratio 1:10), stirred mechanically in a 65 °C water bath for 6 hours, and filtered and washed with distilled water until neutral.

The synthesis of SAP_2_: We weighed a certain amount of pretreated WMR powder and added into a 250 mL three-necked flask under a 70 °C water bath, and then we dispersed it evenly in 30 mL of ultrapure water via mechanical stirring; then, we added the partially neutralized AA, DAAM, PGS, and MBA into the reaction vessel after 40 min, and then we kept the whole reaction under nitrogen protection. After 30 min, the initiator APS was added and the reaction conditions were maintained to complete the polymerization. The reaction products were washed with anhydrous ethanol, cut into pieces, and placed in oven at 65 °C. Finally, the products crushed to obtain SAP_2_ at a constant weight.

SAP_1_ was synthesized in the same way as SAP_2_, except that PGS was not added in the synthesis process.

The structure, morphology, and thermodynamic properties of two kinds of superabsorbent resins that were made in the laboratory were characterized by FTIR, SEM, and TGA.

### 2.2. Characterization of Superabsorbent Polymer

#### 2.2.1. Fourier Transform Infrared Spectroscopy

The sample to be tested and potassium bromide were put into a mortar for grinding in accordance with a certain mass ratio (1:100) to mix the sample and potassium bromide evenly [24]. Then, the sample was made with a tablet pressing machine. FTIR spectra were taken over the range from 4000 to 400 cm^−1^ using a Bracher EQUINX55 FTIR Spectrometer [25].

#### 2.2.2. Morphology Analysis

The dried sample was put into an anhydrous ethanol solution in the centrifuge tube and dispersed evenly by ultrasound for 10 min. Then it was dropped on the copper foil attached to the sample table and dried for later use. Before the test, the sample’s surface was sprayed with gold, and then the sample table was placed into a scanning electron microscope with an accelerating voltage of 5.0 kV to observe its morphological characteristics and pore morphology.

#### 2.2.3. Thermal Gravimetric Analysis of Superabsorbent Polymer

Dry samples of four different superabsorbent polymers were sampled at about 15 mg and placed in a thermal gravimetric analyzer (TGAQ100, City of New Castle, DE, USA) of the American TA Company for testing. The TGA analysis conditions were as follows. The temperature was raised from 0 to 600 °C at a heating rate of 10 °C min^−1^ under a nitrogen atmosphere, wherein the nitrogen flow rate was 20 mL min^−1^.

Before the start of the experiment, we chose healthy seeds. The amount of water-retaining agent to be added was calculated based on the water holding capacity of the sand.

### 2.3. Experiment Design

The experiment was designed to be completely randomized. There were 5 experiments (treatments A, B, C, D, and E), and each was replicated 3 times. The water holding capacity of the sand (Tengger desert) was measured before the start of the experiment, the weight of the sand in each container was weighed, and the amount of the superabsorbent polymer to be applied was calculated from the weight of the sand. We sprinkled an appropriate amount of superabsorbent polymer into the sand and mixed them well. Then, we added an appropriate amount of water. Treatment E was the control group, as you can see from Table 1, as it had no superabsorbent polymer, only water. Then, we planted 30 seeds per group, and the weighed sand and superabsorbent polymer were mixed evenly and spread in an open box of 256 mm × 184 mm × 45 mm. The seeds were buried at a depth of 1 cm from the surface of the sand. The 30 seeds were evenly distributed throughout the box, and the calculated water quality was added. The entire emergence experiment was done in an artificial climate chamber. The emergence environment data required for the specific emergence experiment are shown in Table 2.

In Table 2, “part one” and “part two” represent the no-light and light phases, respectively, and the “0” and “4” represent the levels of light intensity in an artificial climate chamber (RQH-280C, Shanghai Kuangbei Industrial Co., Shanghai, China).

### 2.4. Determination of the Project

#### 2.4.1. Balanced Swelling Rate Test of Superabsorbent Polymer in Distilled Water and Tap Water

The water absorption capacity of a superabsorbent polymer is expressed by its equilibrium water absorption ratio. At room temperature, a certain amount of dry sample with four different water retaining agents was weighed, and then the sample was immersed in excess distilled water to achieve a swelling balance. Finally, excess moisture was filtered through a gauze bag, the weight of the swelling balance superabsorbent polymer was weighed and recorded, and the equilibrium water absorption ratio was calculated using the following formula:

(1) $$Q_{eq}{=(w}_{2}{-w}_{1}{)/w}_{1}$$

where *w*_1_ is the weight before sample processing, *w*_2_ is the weight at which the sample reaches the swelling equilibrium, and *Q_eq_* is the sample equilibrium water absorption ratio.

#### 2.4.2. Seed Emergence Rate and Emergence Potential

The seed emergence rate is calculated by the following formula:

(2) $$ER\left(\%\right)=E_{ter}/T_{er}\times 100$$

where *GR* represents the emergence rate of the seed, *E_ter_* represents the number of germinated seeds at time t, and *T_er_* represents the total number of test seeds.

Emergence potential refers to the percentage of the number of emergence seeds in the tested sample when the number of emergence seeds reached its peak daily during the emergence process. Generally, the percentage of the number of emergence seeds in the first 1/3 period of the specified period of emergence is taken as the standard [26]. It is calculated by the following formula:

(3) $$EP\left(\%\right)=E_{tep}/T_{ep}\times 100$$

where *EP* represents the emergence potential, *E_tep_* is the number of emergence seeds that reached their peak daily within the specified time, and *T_ep_* is the total number of tested seeds.

#### 2.4.3. Water Consumption

The sum of the mass of the sand, the amount of water held by the sand, the maximum amount of water absorbed by the superabsorbent polymer, and the mass of the seeds was taken as the initial mass. The difference between the mass measured at the same time interval and the corresponding initial mass was the amount of water added—that is, the amount of water consumed—when performing the emergence experiments. Because of the different swelling rates between different superabsorbent polymers, the amount of water added each time was different. Thus, we evaluated the water retention capacity of the superabsorbent polymer based on the water consumption and the weight of the added water.

#### 2.4.4. Root length, Seedling Height, and Biomass

The root length and stem length of *Artemisia desertorum* seedlings were measured and recorded before the end of the experiment, and then fresh weight was measured. The seedlings were dried in an oven (105 °C) for 8 h, and the dry weight was weighed again. The oven was produced by Shanghai Experimental Instrument Factory Co., Ltd. (Shanghai, China) Model 101A-1.

#### 2.4.5. Data Statistics and Analysis

The statistical analysis of the experimental data was performed using the SPSS19 software (IBM, Armonk, NY, USA). The effects of different experimental treatments were compared by an ANOVA, and the results were analyzed by Duncan’s test (*p* < 0.05).

## 3. Results and Discussion

### 3.1. FTIR Analysis

**Figure 1:**
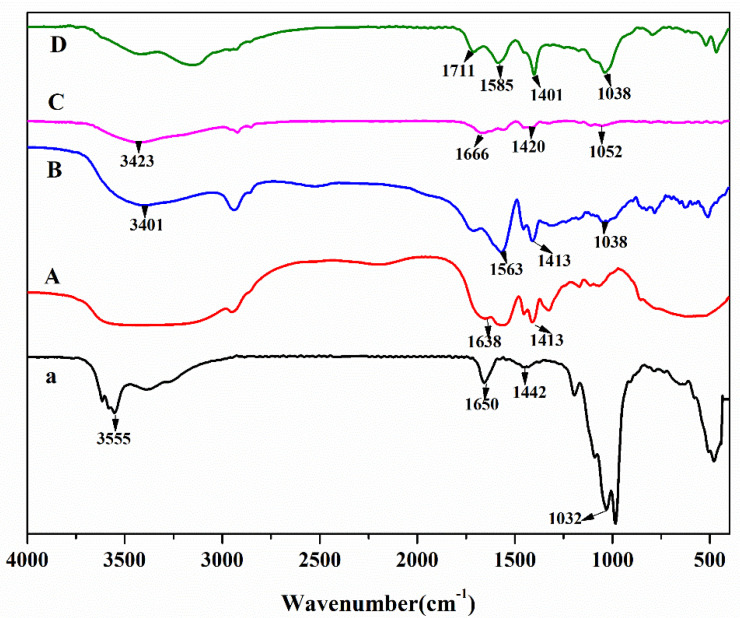
FTIR spectra of (**a**) palygorskite clay, (**A**) treatment A, (**B**) treatment B, (**C**) treatment C, and (**D**) treatment D.

The FTIR spectra of palygorskite clay, treatment A, treatment B, treatment C, and treatment D are shown in Figure 1a–D, respectively. They were bought from the market. C contained C=C (1666 cm^−1^) and –OH (3423 cm^−1^); D contained C=O (1711 cm^−1^), –CONH– (1585 cm^−1^), and C=C (1401 cm^−1^). In Figure 1a, the characteristic absorption peaks at 1032, 1650, and 3555 cm^−1^ are consistent with the characteristic absorption of the Si–O stretching vibration, C=C stretching vibration, and –OH bending vibration of palygorskite clay. The C=C stretching vibration peaks at 1650 and 3555 cm^−1^ and the –OH bending vibration peak almost disappeared after the reaction, while the strong absorption peak caused by the Si–O stretching vibration of palygorskite clay at 1032 cm^−1^ significantly weakened after the reaction, as shown in Figure 1B. The weak absorption peaks of WMR at 1038 and 1638 cm^−1^ were the stretching vibration of C–O (H) and the bending vibration of the –OH group, respectively, as shown in Figure 1A,B, because treatments A and B contained the WMR raw material. As shown in Figure 1A, the characteristic peaks of palygorskite clay were nonexistent, but the characteristic peaks of watermelon rind did exist. The absorption peaks at 1563 and 1413 cm^−1^ in Figure 1B represent carboxyl (–COO–) and C=C groups, indicating a successful reaction between the cross-linking agent and the –OH group of palygorskite clay. The disappearance or weakening of these characteristic peaks indicated the successful grafting of watermelon rind and acrylic acid, as well as the successful preparation of WMR-p (AA–*co*–DAAM) and WMR-p (AA–*co*–DAAM)/PGS.

### 3.2. Scanning Electron Microscope Analysis

**Figure 2:**
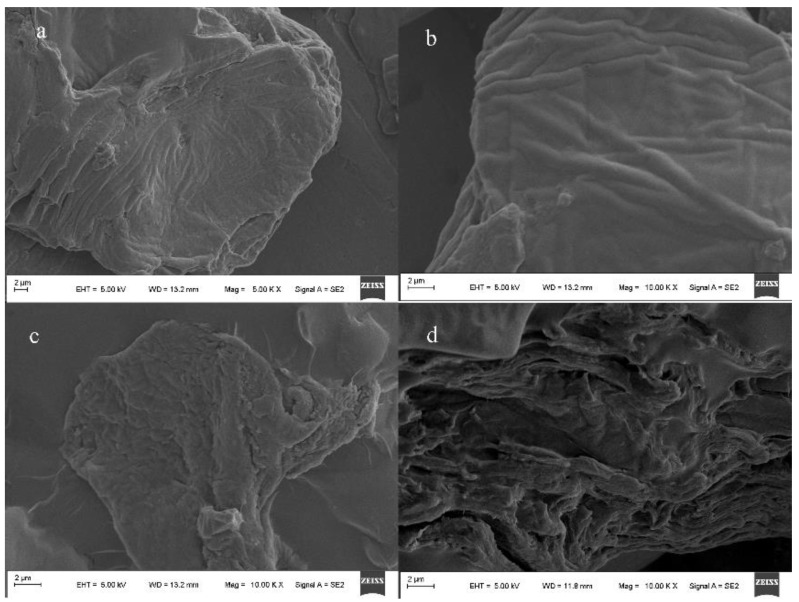
Scanning electron micrographs of WMP (**a**,**b**), SAP_1_ (**c**), and SAP_2_ (**d**).

The surface morphology and structure of an SAP is one of the factors that affects water absorption. Figure 2 shows the SEMs of the surface morphology of the super absorbent resin and the watermelon rind. Figure 2a shows that the surface of the raw material was folded, Figure 2b shows that the surface of the raw material was smooth. Figure 2c,d shows that the surface of the SAP increased its folds and became loose and porous. Studies have shown that a material with a higher porosity has a larger specific surface area, which can increase the contact area between water and polymer networks and can help to expand and improve the water absorption performance of an SAP.

### 3.3. Thermal Gravimetric Analysis

**Figure 3:**
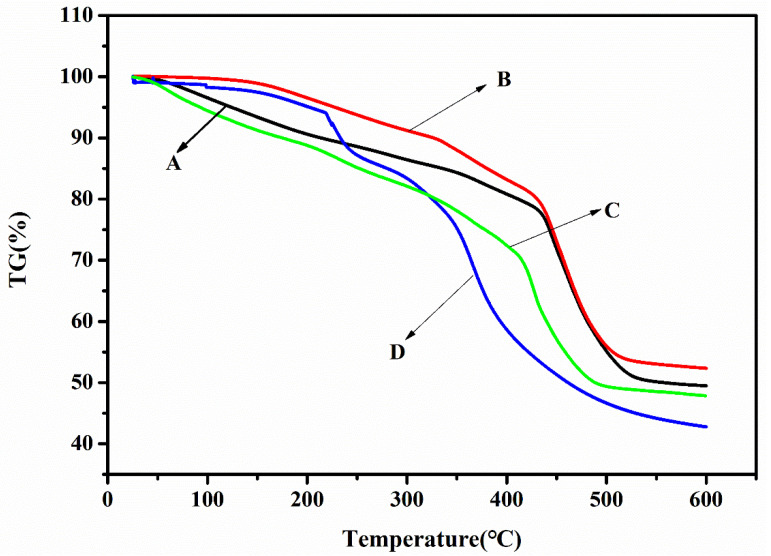
TGA of (**A**) treatment A, (**B**) treatment B, (**C**) treatment C, and (**D**) treatment D. TG: the percentage of the residual mass.

Thermal analysis curves were obtained from the TGA of different SAPs. It can be observed from Figure 3 that the decomposition process of an SAP is a multi-step process. First, from the thermal gravimetric analysis curve, the mass loss of the sample between 0 and 250 °C due to the volatilization of other small molecular compounds or moisture can be seen [27]; compared to treatments A, C, and D, treatment B led to a lesser loss of mass. The mass loss in the temperature range of 250–400 °C may have come from the elimination of carbon dioxide molecules in the polymer chain, and the mass loss of treatment D at this stage was much greater than that of treatments A, B, and C because the maximum degradation temperature of treatment D was 364 °C and the maximum degradation temperatures of treatments A, B, and C were 446, 448, and 430 °C, respectively. The mass loss near this maximum degradation temperature could be attributed to the elimination of water molecules from the two adjacent carboxylic acid groups in the polymer chain to form anhydride molecules [28], resulting in polymer backbone cleavage and the decomposition of the cross-linked network structure [29]. Finally, when the temperature exceeded 500 °C, the most remaining mass was found in treatment B, and treatment D had the least remaining mass. Therefore, the thermal stability of SAP_HDB_ and SAP_RX_ was not as good as SAP_1_ and SAP_2_, which were made in the laboratory.

### 3.4. Water Absorption Capacity of Four Different Superabsorbent Polymers

**Figure 4:**
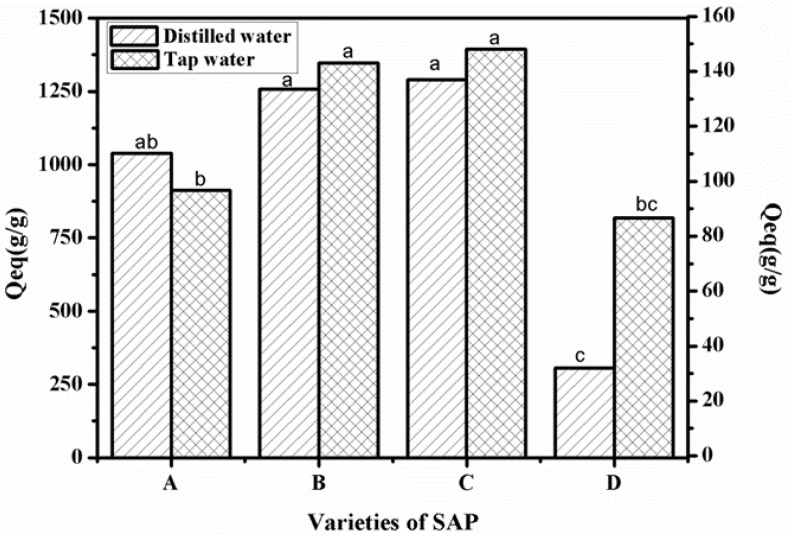
Water absorbency of different SAPs—(**A**) SAP_1_, (**B**) SAP_2_, (**C**) SAP_RX_, and (**D**) SAP_HDB_—in distilled and tap water.

The water absorption capacity of the different superabsorbent polymer can be seen in Figure 4. The water absorption ratios of treatments B and C in tap and distilled water were higher than those of treatments A and D. The water absorption capacity of the superabsorbent polymer was better. This means that when smaller amounts of SAP are required in an actual application process, the process becomes more environmentally friendly and conducive to reducing costs.

### 3.5. Emergence Rate

**Figure 5:**
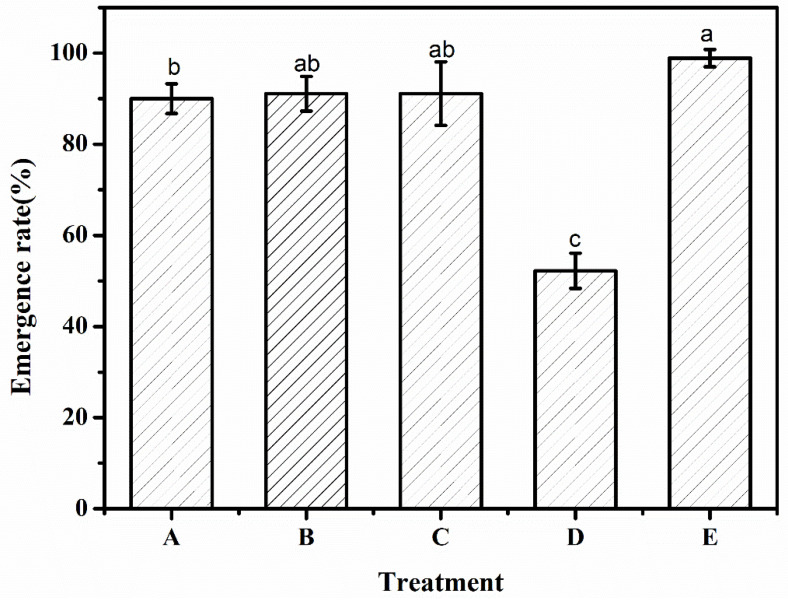
Emergence rate under different experimental treatments—A: SAP_1_; B: SAP_2_; C: SAP_RX_; D: SAP_HDB_; and E: water. Each value represents the mean ± SE (*n* = 30). The different letters indicate significant differences (*p* < 0.05) among treatments, as determined by Duncan’s test.

It can be seen from Figure 5 that the types of superabsorbent polymers were different, as were the emergence rates. Compared with control group E (98.9%), the emergence rates of *Artemisia desertorum* for treatments A, B, C, and D were 90.0%, 91.1%, 91.1%, and 52.2%, respectively, which were slightly lower than the control group. There was no significant difference between treatments B and C (*p* > 0.05), and there was a significant difference (*p* < 0.05) between treatment A and the others. In the experiment, the highest emergence rate of treatment E was 98.9%, which indicated that the quality of the *Artemisia desertorum* seeds was good. Meanwhile, treatments B and C had no significant difference, and the superabsorbent polymer of these treatments had no obvious inhibitory effects on the seed emergence or growth of *Artemisia desertorum* [30]. Meanwhile, treatments A and C showed significant differences, indicating that the superabsorbent polymer of treatments A and C hurt the emergence of *Artemisia desertorum*. This also proved that the superabsorbent polymer prepared in the laboratory with the addition of palygorskite clay is practically applicable to aiding the seed emergence of *Artemisia desertorum*. The experimental data of emergence rate was shown in Table 3.

### 3.6. Emergence Potential

**Figure 6:**
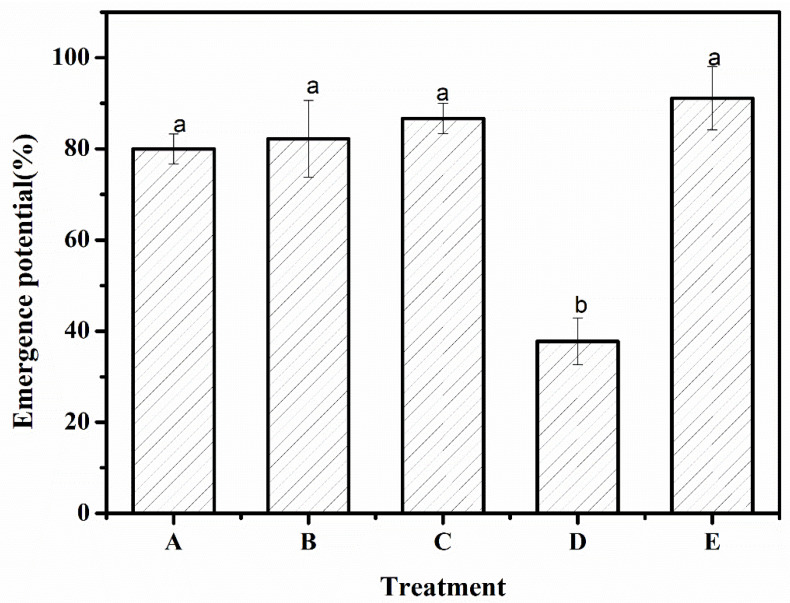
Emergence potential under different experimental treatments—A: SAP_1_; B: SAP_2_; C: SAP_RX_; D: SAP_HDB_; and E: water. Each value represents the mean ± SE (*n* = 30). The different letters indicate significant differences (*p* < 0.05) among treatments, as determined by Duncan’s test.

The results showed that there were significant differences in the emergence potential of *Artemisia desertorum* under different experimental treatments, as shown in Figure 6. The emergence potentials of treatments A, B, and C were 80.0%, 82.2%, and 86.7%, respectively, which were slightly lower than the control treatment group E (*p* > 0.05), while the emergence potential of treatment D was 37.7%, which was significantly lower than the control group with a significant difference (*p* < 0.05). This indicated that there was no significant difference in the emergence speed and uniformity of *Artemisia desertorum* seeds in treatments A, B, C, and E. The possible reasons for the emergence potentials of treatments A, B, C, and D being lower than that of control group E was that in the early stage of the emergence of the *Artemisia desertorum* seeds, the water was absorbed by the dry superabsorbent polymer and the *Artemisia desertorum* seeds together. The water absorbed by the superabsorbent polymer was fixed in its three-dimensional network structure and then slowly released, which made the seeds of *Artemisia desertorum* slowly obtain the moisture required for emergence in the early stages, thus prolonging the emergence time; however, this did not affect the normal emergence of the seeds.

Therefore, the laboratory-prepared SAP_1_ and SAP_2_ had no adverse effects on the normal emergence of *Artemisia desertorum* seeds. The experimental data of emergence potential was shown in Table 3.

### 3.7. Water Consumption

**Figure 7:**
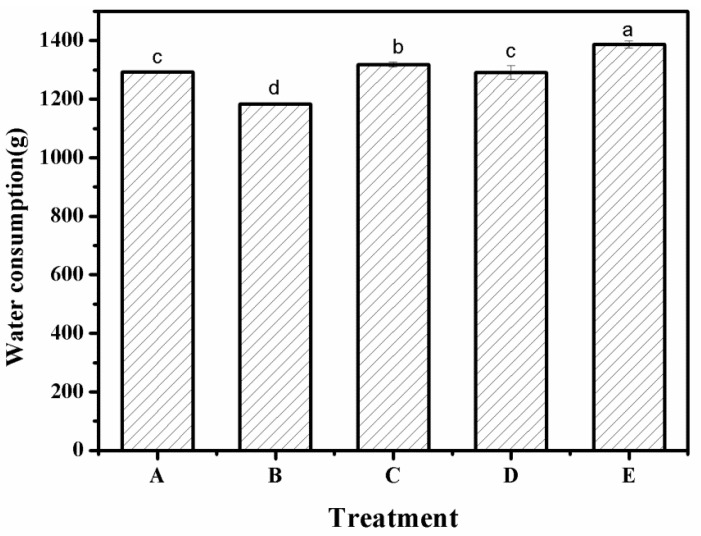
Water consumption under different experimental treatments—A: SAP_1_; B: SAP_2_; C: SAP_RX_; D: SAP_HDB_; and E: water. Each value represents the mean ± SE (*n* = 30). The different letters indicate significant differences (*p* < 0.05) among treatments, as determined by Duncan’s test.

The water consumption of *Artemisia desertorum* after applying different superabsorbent polymers is shown in Figure 7. According to the figure, there were significant differences in the water consumption of *Artemisia desertorum* after applying different superabsorbent polymers. The water consumption of control group E was the highest at 1386.9 g, while the water consumption levels of treatments A and D were 1292.6 and 1291.3 g, respectively. There was no significant difference (*p* > 0.05) between treatments A and D, but treatment B (1184.5 g) had the lowest water consumption with a significant difference (*p* < 0.05) with other treatment groups. Overall, the water consumption of treatment C (1317.6 g) was significantly higher than the other treatments (*p* < 0.05), that is, SAP_RX_’s ability to retain moisture in the sand was weaker than other SAPs. From this result, we could see that the use of a superabsorbent polymer could effectively reduce water consumption, additionally proving the previous conclusion that a superabsorbent polymer has better water absorption and water retention properties than soil by itself. During the emergence and growth of the *Artemisia desertorum* seeds, the water absorbed by the polymer is slowly released, which improves the water use efficiency [31]. Compared with the control group, the application of a superabsorbent polymer led to the consumption of less water; the main reason for this was that some of the moisture absorbed by the superabsorbent polymer was fixed in the three-dimensional network structure of the superabsorbent polymer, so during the growth of *Artemisia desertorum*, it was not directly evaporated and therefore played a good role in water retention [21]. In the growth stage of *Artemisia desertorum*, when the environmental moisture was evaporated and absorbed, the water fixed in the superabsorbent polymer was slowly released to supply the emergence and growth of the plant. Therefore, under the same conditions, the addition of a superabsorbent polymer could effectively reduce the water consumption of *Artemisia desertorum* [32].

### 3.8. Root Length and Stem Length of Different Treatment Groups

**Figure 8:**
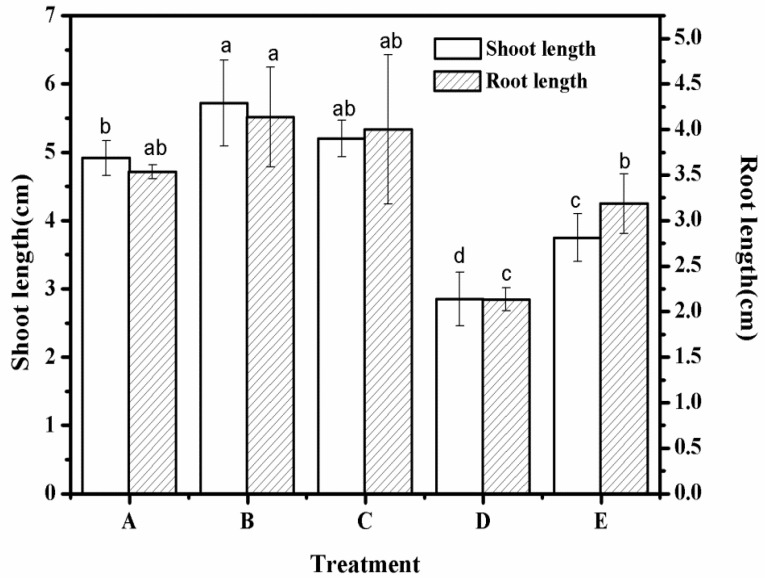
Root length and shoot length under different experimental treatments—A: SAP_1_; B: SAP_2_; C: SAP_RX_; D: SAP_HDB_; and E: water. Each value represents the mean ± SE (*n* = 30). The different letters indicate significant differences (*p* < 0.05) among treatments, as determined by Duncan’s test.

As shown in Figure 8, there were differences between different processing groups. Specifically, the stem lengths of *Artemisia desertorum* for treatments A, B, and C were 4.92, 5.72, and 5.2 cm, respectively, which were significantly higher than that of control group E (*p* < 0.05). Among them, the stem length of treatment B was the highest, and it had no significant difference with that of treatment C (*p* > 0.05). There were significant differences between treatments A, D, and E (*p* < 0.05), though those these differences were significantly lower than those between treatments A, B, C, and E (*p* < 0.05). From this result, it could be seen that compared with control group E, SAP_1_, SAP_2_, and SAP_RX_ all had significant promoting effects on the stem length of *Artemisia desertorum* [33].

The root lengths of *Artemisia desertorum* were different with different superabsorbent resins. The root length of treatment group B was 4.12 cm, which was significantly higher than control group E (*p* < 0.05), while the root lengths of treatments A and C were 3.52 and 3.91 cm, respectively, compared to control group E (3.20 cm); this was not a significant difference (*p* > 0.05). For treatment D, the root length of *Artemisia desertorum* was 2.12 cm, which was significantly lower than the other treatment groups (*p* < 0.05).

It can be seen from Figure 8 that the root length of treatment B with SAP_1_ was the longest. Therefore, SAP_1_ could significantly promote the root length of *Artemisia desertorum* in the growth process, but SAP_2_ and SAP_RX_ were found to have no obvious effects on the growth process. SAP_HDB_ harmed the root length of *Artemisia desertorum*, which may have been caused by different types of ions and their hydrophilic groups contained in the different superabsorbent polymers. Some ions can stimulate the growth of roots and some can harm the growth of roots because the ion concentration is too high. Watermelon rind as a biomass material contains a certain amount of sugar or minerals because fresh watermelon and palygorskite clay contain traces of elements such as calcium, iron, and magnesium. These are required for plant growth and, to a certain extent, promote plant growth. However, super absorbent polymers purchased from markets are all chemically synthesized, with a single element (such as nitrogen) at a high concentration, which does not significantly promote and may even inhibit the growth of plants [34]. SAP_2_ was found to have a very favorable effect on the root growth of *Artemisia desertorum* seedlings. The experimental data of root length and shoot length were shown in Table 3.

### 3.9. Aboveground Biomass, Belowground Biomass and Total Biomass in Different Treatment Groups

**Figure 9:**
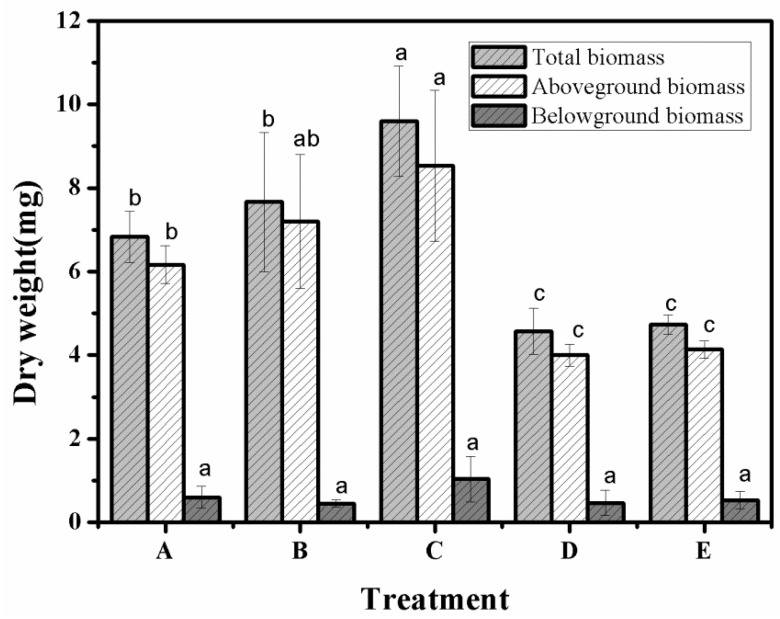
Belowground biomass, aboveground biomass and total biomass under different experimental treatments—A: SAP_1_; B: SAP_2_; C: SAP_RX_; D: SAP_HDB_; and E: water. Each value represents the mean ± SE (*n* = 30). The different letters indicate significant differences (*p* < 0.05) among treatments, as determined by Duncan’s test.

The aboveground biomass accumulation, belowground biomass accumulation, and total biomass accumulation in different treatment groups are shown in Figure 9. As can be seen from the figure, the total biomass of treatment C was 9.6 mg, which was significantly higher than the other treatment groups. Additionally, the differences between treatments A, B, and E were significant (*p* < 0.05), and the biomass values of treatments A and B were significantly lower than that of treatment C and significantly higher than that of control group E. At the same time, the difference between the aboveground biomass and total biomass of treatments B and C was not significant (*p* > 0.05), and the differences between the other treatment groups were consistent with the total biomass. As can be seen from the figure, there was no significant difference between the belowground biomass of the different treatments and control group E (*p* > 0.05).

Based on the determination of aboveground biomass and total biomass accumulation, it was found that the addition of SAP_1_, SAP_2_, and SAP_RX_ significantly promoted the accumulation of the aboveground biomass of *Artemisia desertorum*. The promoting effect of treatment B was the most obvious, which also showed the longest stem length. This was mainly because developed lateral roots can provide nutrients for the growth of aboveground parts from multiple sources [35]. However, the effect of lateral roots on the accumulation of belowground biomass is not obvious because such biomass is predominantly influenced by climate and soil, in addition to root traits [36,37]. Aboveground environments are more exposed to air, so there were differences in aboveground biomass but not belowground biomass between treatment groups. The aboveground biomass of treatment C was higher than that of other groups because it formed the main root—the more developed a main root, the larger biomass of its root system [38,39]. Therefore, the laboratory-made SAP was found to have no inhibitory effects on the biomass accumulation of *Artemisia desertorum*, but it was found to have a certain promoting effect, which indicated that the SAP made in this laboratory is practical. The experimental data of total biomass, aboveground biomass and belowground biomass were shown in Table 3.

## 4. Conclusions

In this paper, an experiment regarding the seed emergence of *Artemisia desertorum* was carried out by using laboratory-made and commercially available superabsorbent polymers. The effects of a superabsorbent polymer on the seed emergence and seedling growth of *Artemisia desertorum* were studied. At the same time, the water absorption, water retention, thermal stability, and salt tolerance of the laboratory-made superabsorbent polymers were studied. We found that the seed emergence and seedling growth of the *Artemisia desertorum* were significantly affected by different superabsorbent polymers. Our laboratory-made SAP_2_ (which had added palygorskite clay in the preparation process) had a better performance than SAP_1_, whether regarding water absorption, water retention, or the promotion of plant growth. SAP_2_ also performed better than SAP_RX_ and SAP_HDB_, and we observed that it had the effect of crust fixation after application; this effect was not observed with SAP_RX_ and SAP_HDB_. SAP_RX_ promoted the biomass accumulation of *Artemisia desertorum* significantly more than other SAPs, which may have been related to its preparation materials. SAP_HDB_ inhibited the emergence and growth of the *Artemisia desertorum* in this experiment, this reason must be further explored. In general, the laboratory-made superabsorbent polymers are low in cost, environmentally friendly, biodegradable, good in moisture retention, have certain promoting effects on the growth of plants, and have no toxic effects on seedlings. Thus a superabsorbent polymer can be applied to the growth process of plants to promote seedling growth, enhance drought resistance, reduce the amount of irrigation needed, and save water resources.

## Figures and Tables

**Table 1:** The experimental design and treatment. SAP: Superabsorbent polymer

Varieties of SAP	Treatment	Sand (g)	Water (g)	SAPs (g)	Source
SAP_1_	A	1535.8	350.2	3.70	Laboratory-Prepared
SAP_2_	B	1535.0	350.0	2.52	Laboratory-Prepared
SAP_RX_	C	1521.9	347.0	2.40	Purchased
SAP_HDB_	D	1536.7	350.4	4.17	Purchased
0	E	1533.3	349.6	0	0

**Table 2:** Setting parameters of the artificial climate chamber in the experiment.

Setting Parameters	Part One	Part Two
Temperature	20 °C	30 °C
Humidity	40%	25%
Light intensity	0	4
Time	720 min	720 min

**Table 3:** The experimental data of emergence rate, emergence potential, root length, shoot length, total biomass, aboveground biomass and belowground biomass for treatments A, B, C, D, and E.

	A	B	C	D	E
Emergence rate (%)	90.0	91.1	91.1	52.2	98.9
Er_1_	3.300	3.811	6.966	3.868	1.905
Emergence potential (%)	80.0	82.2	86.6	37.7	91.1
Er_2_	3.300	8.404	3.350	5.085	6.966
Root length (cm)	3.52	4.12	3.98	2.12	3.17
Er_3_	0.076	0.548	0.822	0.126	0.325
Shoot length (cm)	4.92	5.72	5.20	2.85	3.75
Er_4_	0.257	0.629	0.265	0.391	0.350
Total biomass (mg)	6.83	7.67	9.60	4.57	4.73
Er_5_	0.611	1.662	1.323	0.551	0.231
Aboveground biomass (mg)	6.17	7.20	8.53	4.00	4.13
Er_6_	0.451	1.609	1.801	0.265	0.208
Belowground biomass (mg)	0.60	0.45	1.03	0.47	0.53
Er_7_	0.265	0.087	0.551	0.306	0.208
